# Differences in risk factors for mortality between T2N1M0 and T3N0M0 lobular breast cancer patients: a comparative study

**DOI:** 10.3389/fphar.2025.1550081

**Published:** 2025-03-11

**Authors:** Longjie Xia, Chunxin Qin, Wei Chen, Kang Chen

**Affiliations:** ^1^ College of Life Science and Technology, Guangxi University, Nanning, China; ^2^ Department of Breast Surgery, Weihai Municipal Hospital, Cheeloo College of Medicine, Shandong University, Weihai, China; ^3^ Galactophore Healthcare Department, The Affiliated Weihai Second Municipal Hospital of Qingdao University, Weihai, China

**Keywords:** T2N1M0, T3N0M0, lung-related disease mortality, radiotherapy, lobular breast cancer

## Abstract

**Objective:**

This study aimed to explore the differences in risk factors for mortality between T2N1M0 and T3N0M0 lobular breast cancer, and investigate the factors associated with non-lobular breast cancer mortality.

**Methods:**

Data from 2,693 T2N1M0 and 1,384 T3N0M0 lobular breast cancer patients from the SEER database (2008–2018) were analyzed. The lobular breast cancer-specific and non-lobular breast cancer mortality were compared using the Kaplan-Meier curve and Log-rank test. The Cox proportional hazards regression analysis was used to determine the risk factors associated with non-lobular breast cancer mortality.

**Results:**

The total survival time showed a significant difference between the T2N1M0 and T3N0M0 groups (p = 0.0011). Statistically significant difference were found in lung-related disease mortality (p = 0.0023), with the survival rate of T2N1M0 higher than that of T3N0M0. Age, surgery, radiotherapy, and chemotherapy were independent factors associated with mortality in lung-related disease patients with both subtypes, and compared with T2N1M0, radiotherapy in T3N0M0 increased the risk of lung-related disease mortality (HR = 2.076, 95% CI: 1.4318–3.011).

**Conclusion:**

The T3N0M0 group had a higher mortality rate from lung-related diseases compared to the T2N1M0 group, and radiotherapy may increase the risk of lung-related disease death in T3N0M0 patients. These findings provide valuable information for treatment strategies for T2N1M0 and T3N0M0 subtypes of patients and assist physicians and patients make better treatment choices.

## 1 Introduction

Lobular breast cancer is a widespread malignancy affecting women globally, with an estimated 2.3 million new cases diagnosed each year (Lei et al., 2021; Sung et al., 2021). Despite remarkable advances in modern medicine, selecting optimal treatment strategies for patients with various stages and subtypes of lobular breast cancer remains a daunting task. One of the challenges lies in the marked heterogeneity observed in the disease’s stage and subtype among afflicted individuals (Galimberti et al., 2018; Goldhirsch et al., 2011).

In particular, identifying appropriate treatment options for patients with stage IIB lobular breast cancer is a challenging issue that requires careful assessment of the benefits and risks. Stage IIB lobular breast cancer is characterized by tumor size larger than 5 cm without lymph node involvement (T3N0M0) or tumor size between 2 cm and 5 cm with 1–3 ipsilateral axillary lymph node metastases (T2N1M0) (Wu et al., 2023). Early detection and selection of the optimal treatment strategy are crucial for the survival and quality of life of patients with this subgroup (Trayes and Cokenakes, 2021). However, the available treatment options and prognosis differ between the two subtypes of patients. Therefore, exploring the clinical and treatment data of these subtypes, analyzing the differences in mortality rates, and investigating their relationships with risk factors for mortality are vital in devising more effective treatment strategies.

This primary objective of this study is to investigate the differences in mortality risk factors between T3N0M0 and T2N1M0 lobular breast cancer patients using clinical and treatment data from the SEER database between 2008 and 2018. Additionally, the study aims to identify risk factors that contribute to increased non-lobular breast cancer mortality. Understanding the differences in mortality risk factors between these two subtypes of patients and identifying potential risk factors can help clinicians better select and optimize treatment strategies, ultimately improving patient outcomes.

## 2 Materials and methods

### 2.1 Patient population and data source

In this study, we utilized the Surveillance, Epidemiology, and End Results (SEER) database established by the National Cancer Institute (NCI) (Daly and Paquette, 2019), which regularly gathers patient demographic information, primary tumor site, disease extent, treatment course, and follow-up data. We excluded patients with incomplete or inconsistent treatment records and those with comorbidities such as end - stage organ failure unrelated to breast cancer or its treatment that significantly affected short - term survival. Then, we identified the T2N1M0 and T3N0M0 subgroups of lobular breast cancer patients between 2008 and 2018 based on clinical criteria.

### 2.2 Statistical analysis

We employed the chi-square test or Fisher’s exact test to compare the clinical characteristics of the T2N1M0 and T3N0M0 subgroups. For the comparison of mortality categories, including overall survival (OS), disease-specific survival (DFS), and survival for cardiovascular and lung disease, we used Kaplan-Meier curves and log-rank tests. After identifying statistically significant mortality categories, we selected clinically meaningful and statistically significant clinical characteristics as adverse prognostic risk factors, and performed univariate and multivariate analyses using Cox proportional hazard regression.

## 3 Results

In this study, a total of 4,077 female lobular breast cancer patients were enrolled, with 2,693 patients in the T2N1M0 stage and 1,384 patients in the T3N0M0 stage. Table 1 presents a comparison of the clinical characteristics of the patients, revealing statistically significant differences between the two subgroups with respect to age, race, marital status, surgical status, radiotherapy, chemotherapy, systemic therapy, and PR characteristics.

**TABLE 1:** Characteristics and comparation between T2N1M0 and T3N0M0 patients.

Characteristics	T2N1M0 (n = 2,693)	T3N0M0 (n = 1,384)	*p* value
Age (yr)			<0.001
<60	1,140 (42.3)	465 (33.6)	
≥60	1,553 (57.7)	919 (66.4)	
Race			0.009
White	2,277 (84.6)	1,210 (87.4)	
Black	269 (10.0)	98 (7.1)	
Other	147 (5.5)	76 (5.5)	
Marital status			0.003
Married	1,531 (56.9)	748 (54.0)	
DSSU	691 (25.7)	334 (24.1)	
Widowed	358 (13.3)	245 (17.7)	
Unknown	113 (4.2)	57 (4.1)	
Laterality			0.403
Left	1,358 (50.4)	677 (49.0)	
Right	1,335 (49.6)	706 (51.0)	
Surgery			<0.001
Yes	2,595 (96.4)	1,281 (92.7)	
NO	98 (3.6)	101 (7.3)	
Radiotherapy			0.039
Yes	1,288 (57.8)	712 (51.3)	
NO	1,405 (52.2)	674 (48.7)	
Chemotherapy			<0.001
Yes	1,524 (56.6)	567 (31.0)	
NO	1,169 (43.4)	817 (59.0)	
Systemic treatment			<0.001
Yes	2,256 (83.8)	1,058 (76.4)	
NO	437 (16.2)	326 (23.6)	
Breast Subtype			0.603
HR-/HER2-	33 (1.2)	16 (1.2)	
HR-/HER2+	12 (0.4)	6 (0.4)	
HR+/HER2-	2,516 (93.4)	1,307 (94.4)	
HR+/HER2+	132 (4.9)	55 (4.0)	
ER			0.878
Positive	2,643 (98.1)	1,360 (98.3)	
Negative	50 (1.9)	24 (1.7)	
PR			<0.001
Positive	2,330 (86.5)	1,123 (81.1)	
Negative	363 (13.5)	261 (18.9)	
HER2			0.221
Positive	144 (5.3)	61 (4.4)	
Negative	2,549 (94.7)	1,323 (95.6)	

Note: Data are presented as N (%) and P values are calculated using chi-square test.

Abbreviations: ER, Estrogen Receptor; PR, Progesterone Receptor; HER2, Human epidermal growth factor receptor-2.

Based on the data presented in Table 2, a significant difference in mortality rates was observed between the T2N1M0 and T3N0M0 subgroups according to the overall survival (OS) indicator (p = 0.002), with a higher mortality rate observed in the T2N1M0 subgroup. However, no significant difference was found between the two groups based on the tumor-specific survival (DFS) indicator (p = 0.59). Furthermore, a statistically significant difference in survival time was observed between the two subgroups (p = 0.023), with a longer survival time observed in the T3N0M0 subgroup. In terms of cause of death, a significant difference in lung disease-related death was found between the two subgroups (p = 0.006), with a higher rate of lung disease-related death observed in the T3N0M0 subgroup.

**TABLE 2:** Comparation of survival outcomes between the T2N1M0 and T3N0M0 groups.

Outcomes	T2N1M0 (n = 2,693)	T3N0M0 (n = 1,384)	*p* value
OS			0.002
Death	439 (16.3)	280 (20.2)	
Alive	2,254 (83.7)	1,104 (79.8)	
DFS			0.59
Death	224 (8.3)	122 (8.8)	
Alive	2,469 (91.7)	1,262 (91.2)	
Survival months	62.67 (23.89)	60.84 (25.00)	0.023
Death cause			0.017
Alzheimers	11 (0.4)	14 (1.0)	0.032
Breast	210 (7.80)	113 (8.2)	0.726
Diabetes Mellitus	4 (0.1)	7 (0.5)	0.053
Gastrointestinal disease	8 (0.3)	6 (0.4)	0.573
Hematological malignancy	3 (0.1)	3 (0.2)	0.415
Infectious	10 (0.4)	1 (0.1)	0.111
Kidney diseases	3 (0.1)	3 (0.2)	0.415
Liver Disease	3 (0.1)	3 (0.2)	0.415
Other Cause	70 (2.6)	41 (3.0)	0.566
Other Malignant Cancer	19 (0.7)	15 (1.1)	0.208
Cardio-cerebrovascular diseases	70 (2.6)	44 (3.3)	0.335
Lung diseases	28 (1.0)	30 (2.2)	0.006

Note: Data are presented as N (%) and P values are calculated using chi-square test.

Abbreviations:OS, overall survival; DFS, disease-specific survival.

**FIGURE 1:**
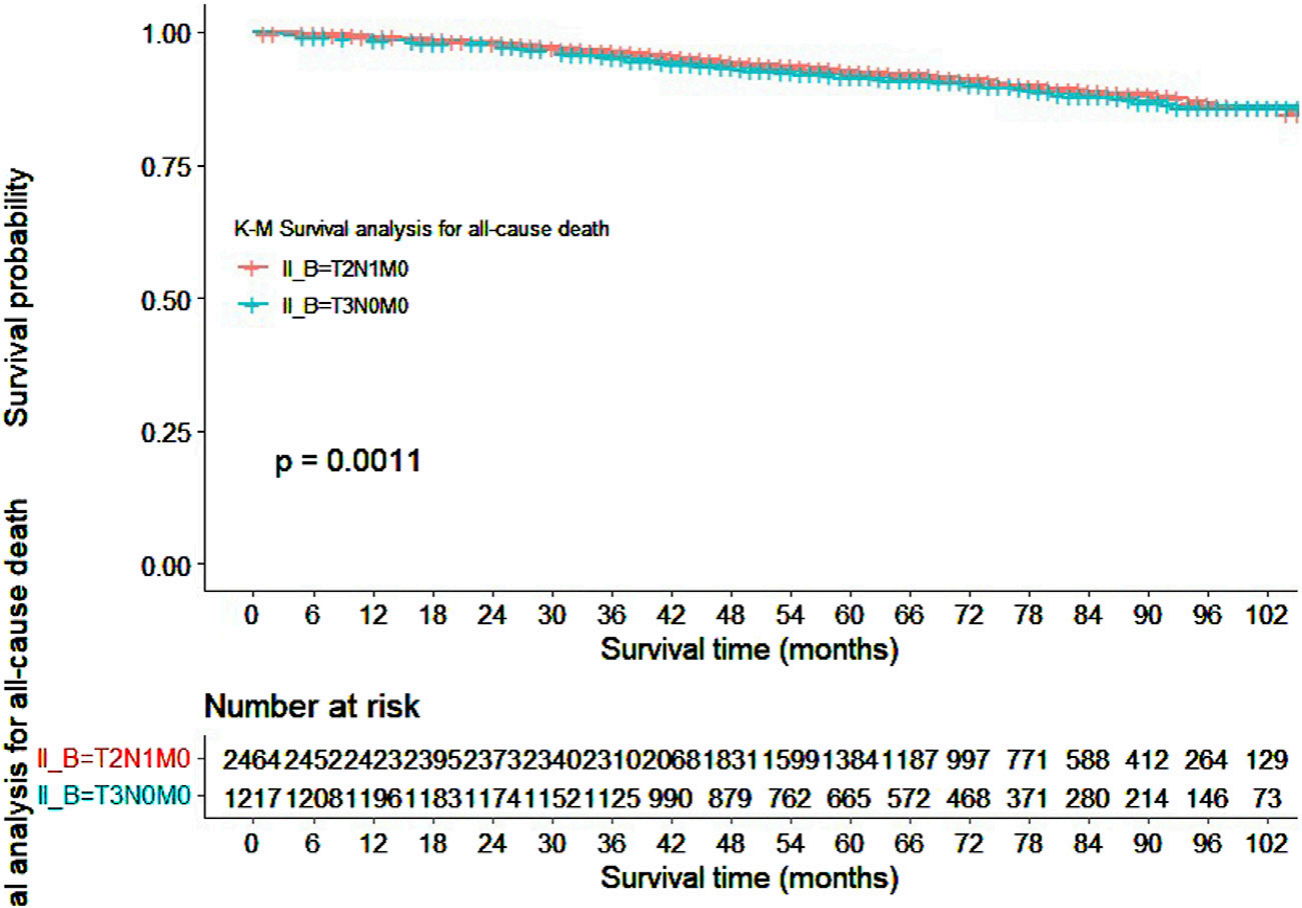
Kaplan-Meier curves for overall survival between the T2N1M0 and T3N0M0 subgroups.

**FIGURE 2:**
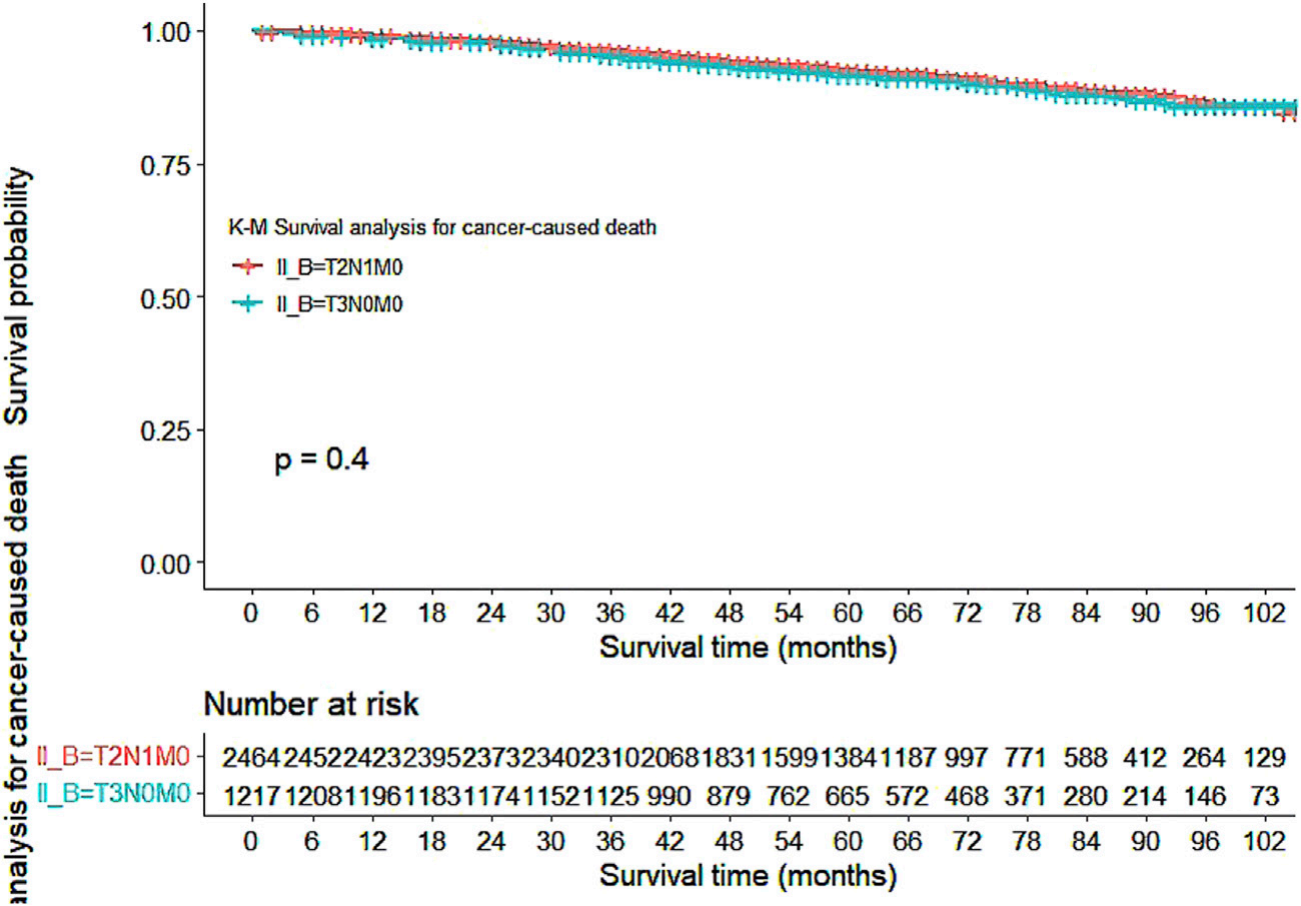
Kaplan-Meier curves for cancer-caused death between the T2N1M0 and T3N0M0 subgroups.

**FIGURE 3:**
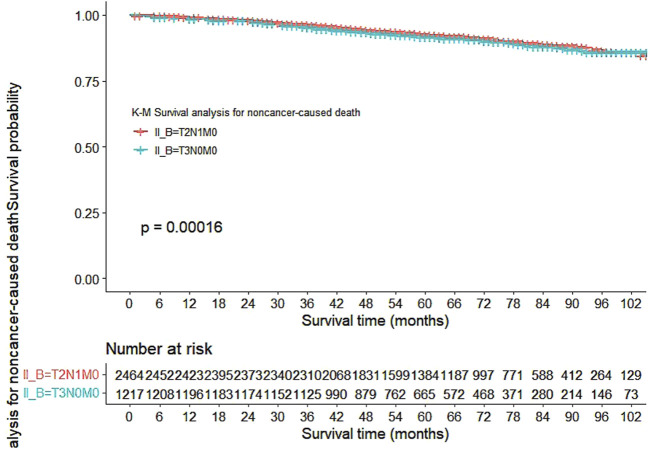
Kaplan-Meier curves for noncancer-caused death between the T2N1M0 and T3N0M0 subgroups.

**FIGURE 4:**
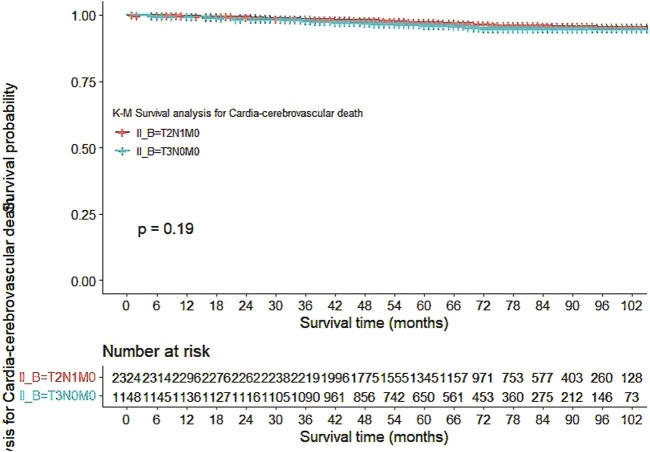
Kaplan-Meier curves for Cardio-cerebrovascular diseases-related death between the T2N1M0 and T3N0M0 subgroups.

**FIGURE 5:**
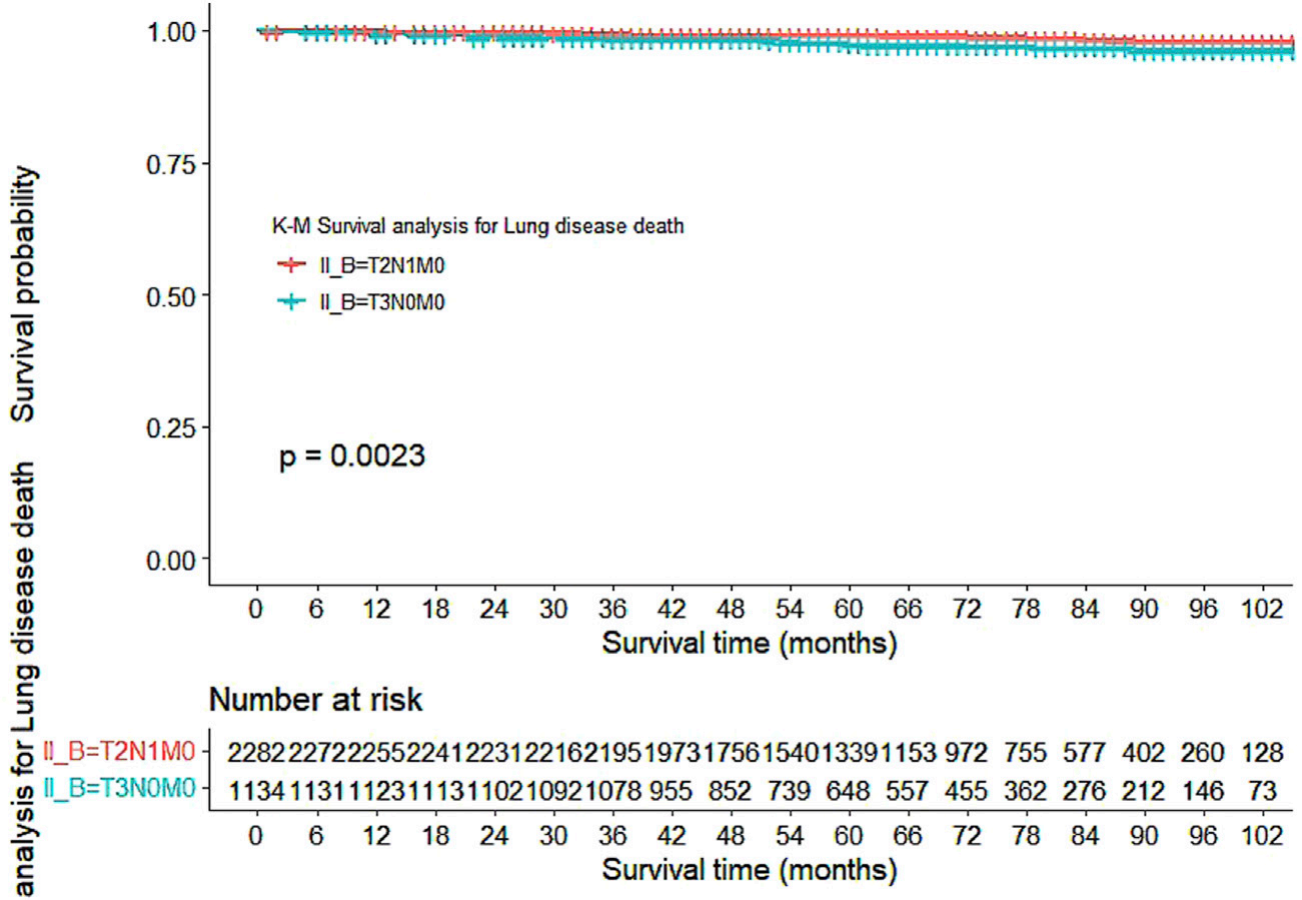
Kaplan-Meier curves for lung disease-related death between the T2N1M0 and T3N0M0 subgroups.

Our study also utilized Kaplan-Meier curves to compare OS, DFS, cardiovascular disease-related death, and lung disease-related death between the T2N1M0 and T3N0M0 subgroups. The results presented in Figures 1–5 showed a significant difference in OS between the two subgroups (p = 0.0011), while there was no significant difference in DFS (p = 0.4). However, there was a significant difference in non-tumor-related mortality (p = 0.00016), with no significant difference in cardiovascular disease-related death (p = 0.19), but a significant difference in lung disease-related death (p = 0.0023). Notably, the T2N1M0 subgroup showed a higher survival rate than the T3N0M0 subgroup.

Table 3 shows the results of the COX proportional hazard regression analysis of lung disease-related death for T2N1M0 and T3N0M0 subgroups, with both univariate and multivariate analyses conducted. The results demonstrated that age (years), surgery, radiotherapy, chemotherapy, and systemic treatment are significant factors associated with lung disease-related death. In the T2N1M0 subgroup, age, surgery, and radiotherapy were significant risk factors, with surgery having the highest hazard ratio (HR) of 4.069, followed by radiotherapy (2.264) and chemotherapy (3.886). In the T3N0M0 subgroup, age, surgery, radiotherapy, and chemotherapy were significant risk factors, with radiotherapy having the highest HR of 3.502, followed by surgery (3.223) and chemotherapy (4.742). Importantly, radiotherapy was found to have a higher risk (HR = 2.076, 95% CI: 1.4318–3.011) in the T3N0M0 subgroup, while the risk of radiotherapy was lower (HR = 1.738, 95% CI: 1.247–1.958) in the T2N1M0 subgroup. These results suggest that age, surgery, radiotherapy, and chemotherapy are independent factors associated with lung disease-related death in lobular breast cancer patients with T2N1M0 and T3N0M0 staging, and radiotherapy carries a higher risk in the T3N0M0 subgroup compared to the T2N1M0 subgroup.

**TABLE 3:** Univariate COX survival analysis and multivariable COX survival analysis For T2N1M0 and T3N0M0 lung disease-related death.

Variable	Univariate COX		Multivariable COX	
	HR (95% CI)	P Value	HR (95% CI)	P Value
T2N1M0 and T3N0M0 lung disease-related death				
Age (years old)	1.099 (1.086–1.113)	<0.001	1.088 (1.073–1.102)	<0.001
Surgery	4.693 (3.074–7.167)	<0.001	2.447 (1.545–3.875)	<0.001
Radiation	1.738 (1.323–2.282)	<0.001	1.342 (1.049–1.847)	0.047
Chemotherapy	3.402 (2.568–4.508)	<0.001	1.290 (0.924–1.802)	0.134
Systemic treatment	2.378 (1.789–3.161)	<0.001	1.182 (0.852–1.639)	0.315
ER	1.031 (0.330–3.222)	0.958	-	-
PR	1.087 (0.7361–1.604)	0.676	-	-
HER2	1.365 (0.701–2.659)	0.360	-	-
T2N1M0 lung disease-related death				
Age (years old)	1.107 (1.097–1.117)	<0.001	1.093 (1.082–1.105)	<0.001
Surgery	4.069 (3.017–5.489)	<0.001	1.875 (1.342–2.619)	<0.001
Radiation	2.264 (1.829–2.803)	<0.001	1.563 (1.247–1.958)	<0.001
Chemotherapy	3.886 (3.082–4.899)	<0.001	1.256 (0.955–1.653)	0.102
Systemic treatment	2.887 (2.354–3.541)	<0.001	1.253 (0.983–1.598)	0.068
ER	1.008 (0.4171–2.435)	0.986	-	-
PR	1.507 (1.172–1.938)	0.001	0.976 (0.7570–1.259)	0.853
HER2	1.193 (0.723–1.967)	0.490	-	-
T3N0M0 lung disease-related death				
Age (years old)	1.117 (1.100–1.134)	<0.001	1.099 (1.081–1.118)	<0.001
Surgery	3.223 (2.106–4.933)	<0.001	1.346 (0.827–2.190)	0.231
Radiation	3.502 (2.475–4.954)	<0.001	2.076 (1.4318–3.011)	<0.001
Chemotherapy	4.742 (3.077–7.308)	<0.001	1.266 (0.7692–2.083)	0.353
Systemic treatment	3.400 (2.509–4.608	<0.001	1.313 (0.9073–1.899)	0.148
ER	0.967 (0.239–3.899)	0.962	-	-
PR	1.886 (1.343–2.650)	<0.001	1.253 (0.8863–1.773)	0.201
HER2	0.905 (0.424–1.929)	0.796	-	-

Abbreviations:ER, Estrogen Receptor; PR, Progesterone Receptor; HER2, Human epidermal growth factor receptor-2.

## 4 Discussion

The study found that T3N0M0 lobular breast cancer patients treated with radiotherapy had a higher incidence of lung disease-related death when compared to T2N1M0 patients. This finding is consistent with previous research indicating a higher risk of lung toxicity in lobular breast cancer patients treated with radiation therapy (Dörr et al., 2005; Vasiljevic et al., 2018; Werner et al., 2019). Age and radiotherapy were found to be independent factors influencing lung disease-related death in T3N0M0 patients, and age was identified as a crucial risk factor for lung toxicity in lobular breast cancer patients (Dörr et al., 2005). These findings suggested that the use of radiotherapy should be approached with greater caution when treating T3N0M0 lobular breast cancer patients, particularly in elderly patients.

In cases where the risk of radiation toxicity is high, alternative treatments such as chemotherapy or targeted therapy may be considered, especially in HER2-positive lobular breast cancer patients (Feng et al., 2019; Masuda et al., 2017). For T3N0M0 patients, although our study didn't clarify radiotherapy’s relation to recurrence risk or pathological type, some research suggests certain subtypes may respond differently. Clinically, its impact on recurrence varies. We must consider these factors when assessing radiotherapy’s risks and benefits. If the recurrence risk is low, radiotherapy’s harm may outweigh its benefits. But for those with higher recurrence risk, it could still be necessary. Meanwhile, some patients like T2N1M0 ones need radiotherapy due to lymph node metastases (Darby et al., 2011; Coles et al., 2017). Hence, personalized treatment plans should be formulated according to the patient’s specific situation, taking into account the potential advantages and disadvantages of each treatment modality.

For patients with T3N0M0 lobular breast cancer, mastectomy is a potential alternative to radiotherapy. The National Surgical Adjuvant Breast and Bowel Project (NSABP) study demonstrated that mastectomy alone, without adjuvant radiotherapy, can provide equivalent local control and survival outcomes as breast-conserving surgery and radiotherapy in patients with T1-T2N0M0 lobular breast cancer (Fisher et al., 2002). While this study did not involve patients with T3N0M0 lobular breast cancer, it implies that mastectomy may be a safe treatment option for certain patient subgroups. Therefore, mastectomy should be considered as a potential option and compared with radiotherapy when devising treatment plans. Another feasible alternative to radiotherapy is targeted therapy. Trastuzumab, a targeted monoclonal antibody against HER2, has been shown to improve survival outcomes in HER2-positive lobular breast cancer patients (Slamon et al., 2001; Tolaney et al., 2015). The Herceptin Adjuvant (HERA) Trial Study Team demonstrated that the risk of local recurrence requiring radiotherapy did not increase in early-stage HER2-positive lobular breast cancer patients treated with trastuzumab (Piccart-Gebhart et al., 2005; Tolaney et al., 2015). Therefore, targeted therapy may serve as a substitute for radiotherapy in some patients.

In addition to exploring alternative treatment options for lobular breast cancer, it is crucial to address the potential risks associated with radiotherapy. The underlying mechanisms of radiation-induced lung toxicity are not yet fully understood, but evidence suggests that inflammation, fibrosis, and vascular damage in lung tissue may contribute to this adverse effect. Patients with pre-existing lung diseases, such as chronic obstructive lung disease (COPD) or interstitial lung disease (ILD), may be at higher risk of developing radiation-induced lung disease, which can worsen their conditions and potentially result in non-tumor-related deaths (Hanania et al., 2019; Arroyo-Hernández et al., 2021). Therefore, it is important to implement various treatment approaches and preventative measures to mitigate this risk (Jacobson et al., 2021; Chen et al., 2021; Han et al., 2019). For instance, a study in patients with non-small cell lung cancer found that the use of oxygen-enhancing agents and propofol could reduce the incidence of lung complications (Han et al., 2019).

Furthermore, in addition to radiation-induced lung toxicity, radiotherapy has also been linked to a range of other long-term side effects, such as cardiovascular disease and secondary malignancies (Chargari et al., 2013; Darby et al., 2013). Although our study did not find an association between radiotherapy and cardiovascular disease-related mortality in T2N1M0 and T3N0M0 lobular breast cancer patients, previous research has shown that radiotherapy increases the risk of cardiovascular disease, especially in those with left-sided lobular breast cancer, and that the pathogenesis of radiation-induced cardiovascular disease may involve radiation-induced injury to the coronary arteries, heart valves, and pericardium (Darby et al., 2013; Sardar et al., 2017).

Although we were unable to incorporate specific clinicopathological features such as tumor grade and Ki - 67 index into our analysis due to the limitations of the SEER database, we recognize their potential significance in breast cancer research. Previous studies have demonstrated that tumor grade is closely associated with the aggressiveness of the tumor, and a higher grade often indicates a poorer prognosis (Stenmark et al., 2023). Similarly, the Ki - 67 index reflects the proliferative activity of tumor cells, with higher values suggesting more rapid cell division and potentially more aggressive disease behavior (Martins-Branco et al., 2023). In the context of our study, while we lack direct data on these factors, it is reasonable to assume that they could interact with the variables we have analyzed, such as the T2N1M0 and T3N0M0 groupings, and further influence the mortality risk factors. In the future, we aim to conduct multi - center prospective studies to include more comprehensive data, prioritizing features like tumor grade and Ki - 67 index, to confirm hypothesized relationships and better understand lobular breast cancer mortality pathophysiology.

Our study highlights the importance of prolonged surveillance and monitoring of lobular breast cancer patients, especially those who have undergone radiotherapy. It is crucial to investigate and manage any discomfort or symptoms in a timely manner to aid in the early identification and intervention of lung ailments, hence mitigating the risk of lung disease-related mortality while enhancing both patient survival and quality of life.

However, our study has several limitations. Firstly, our sample size is relatively small, necessitating larger-scale studies to validate our findings. Secondly, our data was obtained from retrospective studies, which may introduce information bias and unaccounted confounding variables. Thirdly, our study only evaluated the risk of lung diseases associated with radiotherapy, and did not consider other potential adverse effects such as breast fibrosis and lymphedema. Lastly, potential factors such as genotypes and molecular subtypes were not taken into consideration, despite their significant influence on treatment approaches and prognosis. Notwithstanding these limitations, although our study focused on T2N1M0 and T3N0M0 lobular breast cancer patients, the identified risk factors, such as radiotherapy - related lung - disease mortality, could still offer valuable insights for other subtypes and stages. Given that different subtypes vary in biology and treatment response, radiotherapy - induced toxicity remains a common concern across the board. This connection between our findings and broader patient groups highlights the potential for further exploration. In future research, we plan to collaborate with multiple institutions to enlarge the sample size. Prospective studies will be carried out by closely following newly diagnosed patients and collecting comprehensive data regularly to better control variables and accurately understand mortality risk factors, aiming to improve treatment strategies.

## 5 Conclusion

In conclusion, our study provides valuable insights into the treatment of lobular breast cancer, especially for T3N0M0 lobular breast cancer patients. The findings indicate that the administration of radiotherapy should be cautiously evaluated in specific patients to optimize their survival and quality of life. Our study provides valuable information for the treatment of T3N0M0 patients, which can assist clinicians in making informed decisions and enhancing the patients’ clinical outcomes.

## Data Availability

The datasets presented in this study can be found in online repositories. The names of the repository/repositories and accession number(s) can be found below: https://seer.cancer.gov/.
